# The complete chloroplast genome sequence of *Horsfieldia amygdalina* (Myristicaceae)

**DOI:** 10.1080/23802359.2019.1688122

**Published:** 2019-11-08

**Authors:** Feng-Liang Zhang, Chang-Li Mao, Xiao-Qin Li, Tian Yang, Yu Wu

**Affiliations:** Yunnan Institute of Tropical Crops, Jinghong, China

**Keywords:** Horsfieldia amygdalina, chloroplast genome, Myristicaceae

## Abstract

*Horsfieldia amygdalina* is a member of Myristicaceae. The *H. amygdalina* chloroplast genome is found to be 155,683 bp in length and has a base composition of A (29.99%), G (19.32%), C (19.92%), and T (30.77%). The genome contained two short inverted repeat (IRa and IRb) regions (37,754 bp) which were separated by a large single copy (LSC) region (86,931 bp) and a small single copy (SSC) region (30,998 bp). The genome encodes 121 unique genes, including 86 protein-coding genes, 27 transfer RNA (tRNA) genes, and 8 ribosomal RNA (rRNA) genes. Further, complete chloroplast sequence of *H. amygdalina* was aligned together with *Horsfieldia pandurifolia*, *Myristica yunnanensis* and other Magnoliales and basal angiosperms species which have reported the complete chloroplast sequence. This complete chloroplast genome will provide valuable information for the development of DNA markers for future species resource development and phylogenetic analysis of *H. amygdalina*.

*Horsfieldia amygdalina*, belongs to Myristica of Myristicaceae, is a tall arbor tree and mainly distributed in Guangdong, Guangxi, Hainan and Yunnan provinces, China, its resources are scarce, the distribution area is limited and belong class II endangered plants in China (Xue and Fang 2012). So far, *H. amygdalina* is reported on fatty acid ingredients (Hu et al. 2010; Xue and Fang 2012; Peng 2017), seed propagation (Hu et al. 2011), leaf traits variation (Hu et al. 2017) and it has been analyzed as the taxonomic group with the other 10 species of Myristicaceae to discuss the taxonomic position of *Horsfieldia pandurifolia* (Wu et al. 2015), but there have been no reports on molecular genetics. In this study, we characterized the complete chloroplast genome sequence of *H. amygdalina* for phylogenetic analysis. The annotated genome sequence has been deposited Genbank under the accession number MK285561.

The fresh leaves of *H. amygdalina* was collected in 2017 from plantation base of Yunnan Institute of Tropical Crops (YITC), Jinghong, China (100°47′E, 22°00′N), and its genome DNA was extracted using the DNeasy Plant Mini Kit (QIAGEN, Valencia, CA). A specimen of this tree was conserved in YITC. Genome sequencing was performed using Roche/454, sequencing libraries were prepared by the GS Titanium library preparation kit. The chloroplast genome assembled using CLC Genomic Workbench v3.6 (http://www.clcbio.com). The genes in the chloroplast genome were predicted using the DOGMA program (Wyman et al. 2004).

The circular genome is 155,683 bp in size, and comprises a large single copy (LSC) region (86,931 bp), a small single copy (SSC) region (30,998 bp), and two short inverted repeat (IRa and IRb) regions (37,754 bp). The base composition of the circular chloroplast genome is A (29.99%), G (19.32%), C (19.92%), and T (30.77%). GC content of 39.24% for the whole *H. amygdalina* chloroplast genome. The *H. amygdalina* chloroplast genome encodes a total of 121 unique genes, including 86 protein-coding genes, 27 transfer RNA genes, and 8 ribosomal RNA genes. There were 30 genes duplicated in the IR regions. The LSC region contained 75 genes, which including 56 protein-coding genes, 17 tRNA genes and 1 rRNA genes whereas 7 protein-coding genes, 4 tRNA genes and 4 rRNA genes were including in the SSC region. The introns were detected in 11 genes, among them, *psbB, rpoB, atpH, rpl23, rps19-fragment, trnQ-UUG, trnS-GGA, trnV-GAC, ndhH, trnL-CAA* have 1 intron and *rps7* gene has 2 introns.

To study *H. amygdalina* phylogenetic relationship with the angiosperms, *H. pandurifolia and Myristica yunnanensis* of Myristicaceae (Changli et al. 2019a, 2019b) and other complete chloroplast genome sequences of angiosperms were download for analyses. The maximum likelihood phylogenetic was performed using MEGA X (Kumar et al. 2018) (Figure 1). A bootstrap analysis was performed on the resulting phylogenetic tree, and values were obtained after 1000 replications. The result shows that *H. amygdalina* was clustered with other species and closely to *H. pandurifolia* and *Myristica yunnanensis* chloroplast complete genome.

**Figure 1.**
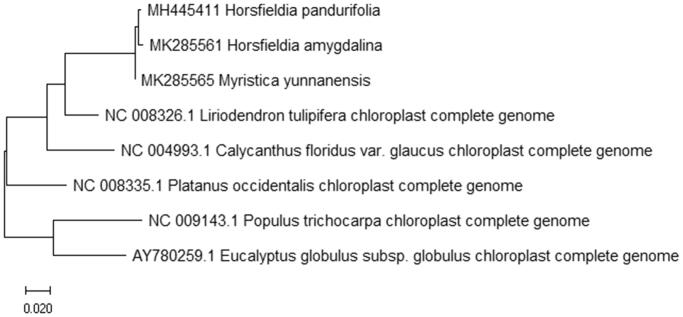
Maximum likelihood phylogenetic tree of *H. amygdalina* with 7 species based on complete chloroplast genome sequences. The gene’s accession number is list in figure and the data of *H. pandurifolia* and *M. yunnanensis* come from author.

The complete chloroplast genome of *H. amygdalina* would provide information on development of molecular markers and phylogenetic analysis in the future.
